# Cellular and Behavioral Effects of Cranial Irradiation of the Subventricular Zone in Adult Mice

**DOI:** 10.1371/journal.pone.0007017

**Published:** 2009-09-15

**Authors:** Françoise Lazarini, Marc-André Mouthon, Gilles Gheusi, Fabrice de Chaumont, Jean-Christophe Olivo-Marin, Stéphanie Lamarque, Djoher Nora Abrous, François D. Boussin, Pierre-Marie Lledo

**Affiliations:** 1 Institut Pasteur, Laboratory for Perception and Memory, Paris, France; 2 Centre National de la Recherche Scientifique (CNRS) Unité de Recherche Associée (URA), Paris, France; 3 CEA, DSV, iRCM, SCSR, Laboratoire de RadioPathologie, INSERM U967, Fontenay-aux-Roses, France; 4 Institut Pasteur, Unité Analyse d'Images Quantitative, CNRS (URA 2582), Paris, France; 5 INSERM U862, Neurocentre Magendie, Neurogenesis and Pathophysiology group, Bordeaux, France; 6 Université de Bordeaux, Bordeaux, France; Chiba University Center for Forensic Mental Health, Japan

## Abstract

**Background:**

In mammals, new neurons are added to the olfactory bulb (OB) throughout life. Most of these new neurons, granule and periglomerular cells originate from the subventricular zone (SVZ) lining the lateral ventricles and migrate *via* the rostral migratory stream toward the OB. Thousands of new neurons appear each day, but the function of this ongoing neurogenesis remains unclear.

**Methodology/Principal Findings:**

In this study, we irradiated adult mice to impair constitutive OB neurogenesis, and explored the functional impacts of this irradiation on the sense of smell. We found that focal irradiation of the SVZ greatly decreased the rate of production of new OB neurons, leaving other brain areas intact. This effect persisted for up to seven months after exposure to 15 Gray. Despite this robust impairment, the thresholds for detecting pure odorant molecules and short-term olfactory memory were not affected by irradiation. Similarly, the ability to distinguish between odorant molecules and the odorant-guided social behavior of irradiated mice were not affected by the decrease in the number of new neurons. Only long-term olfactory memory was found to be sensitive to SVZ irradiation.

**Conclusion/Significance:**

These findings suggest that the continuous production of adult-generated neurons is involved in consolidating or restituting long-lasting olfactory traces.

## Introduction

Neurocognitive deficits and olfactory changes are frequently observed after chemotherapy and cranial radiotherapy in adult patients [1], [2]. These changes may result from damage to the neural stem cell (NSC) populations of the subgranular zone of the dentate gyrus (DG), the hippocampus and the subventricular zone (SVZ) lining the forebrain lateral ventricles [3], [4]. NSCs continually generate new neurons, which are recruited to the DG and the olfactory bulb (OB) of adult mammals [5]. New neuronal progenitors generated in the SVZ migrate along the rostral migratory stream (RMS) towards the OB. Within the OB, they integrate into the granule cell layer (GCL), the external plexiform layer (EPL) or the glomerular layer (GL), giving rise to both gamma-aminobutyric acid (GABA)- and dopamine-containing interneurons [6]–[10]. This ongoing neurogenesis is essential for maintenance of the integrity of the OB circuitry. The blocking of this process depletes the population of OB interneurons [11].

Activity-dependent factors regulate OB neurogenesis, suggesting that adult neurogenesis is not exclusively constitutive [12]–[15]. The new cells added to the OB and DG circuits undergo functional integration [16], [17], and this process is thought to be important for learning and memory [5]. Spatial memory deficits have been observed following the disruption of neurogenesis in transgenic mice [11], [18], [19]. However, the functional relevance of adult neurogenesis in olfaction remains unclear. Some studies have suggested that new neurons are not required for olfaction, whereas others have implicated adult-generated neurons in a number of olfactory functions. For example, olfaction has been shown to be unaffected in Bax-knockout mice [20] and in mice producing a neuron-specific enolase-diphtheria toxin [11], despite the significantly lower than normal level of neurogenesis in both transgenic models. By contrast, both mice lacking neural cell-adhesion molecule (NCAM) and mice with the brain-derived neurotrophic factor (BDNF) Val66Met knock-in display impaired OB neurogenesis and odor discrimination [21], [22]. Olfactory discrimination is also impaired in aging rodents, mice heterozygous for leukemia inhibitory factor receptor (Lifr +/−), and waved-1 mutant mice (a hypermorph of TGF-alpha), in which OB neurogenesis level is reduced [23]. Finally, a correlation has been found between the degree of OB neurogenesis and olfactory memory [24], [25], providing further support for the hypothesis that adult neurogenesis plays an important role in olfaction.

These inconsistencies may result from the use of different ablation techniques, affecting both the DG and OB regions. Precise techniques for disrupting adult neurogenesis in specific areas of the brain may make it possible to assign behavioral functions to each neurogenic system. Focal irradiation of the SVZ leads to a dose-dependent loss of cell types in this region and repopulation may take several months [26]. Here, we used focal SVZ irradiation in adult mice to disrupt the production of new OB neurons without affecting the rest of the brain or the body. We examined the functional effects of irradiation on odorant detection, discrimination and olfactory memory. We found that the continuous recruitment of adult-generated OB neurons was not required for any of the olfactory functions tested except for long-term olfactory memory, which was less robust after irradiation.

## Results

### Focal irradiation of the adult SVZ strongly reduced OB neurogenesis

**Figure 1:**
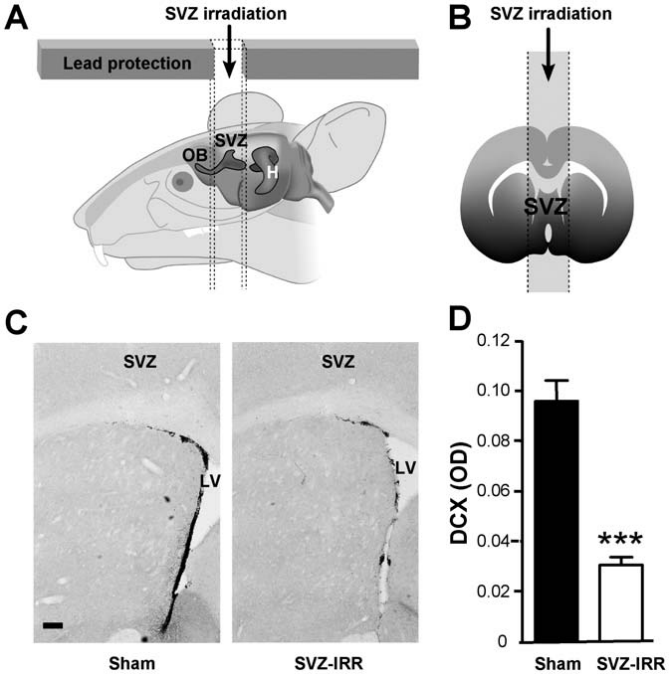
Focal irradiation decreased DCX immunoreactivity in the SVZ. (A, B) Focal gamma-ray irradiation of the SVZ. Adult mice were anesthetized and placed in a stereotaxic frame for cranial irradiation. A lead shield protected their body during exposure of the SVZ to gamma rays. A total dose of 15 Gray was delivered in three equal fractions administered at two-day intervals. H, hippocampus. (C) DCX staining of neuroblasts in a coronal section of the SVZ from a sham-treated mouse (left) and from an irradiated mouse 7 months after SVZ irradiation (right). Note the weaker DCX staining in the SVZ of the irradiated (IRR) mouse. LV, Lateral ventricle. (Scale bar: 100 µm.). (D) Densitometric analysis of DCX immunoreactivity in the SVZ of sham-treated and irradiated mice 7 months after irradiation. OD, optical density. Student's *t* test; *** *p*<0.0001 (*n* = 6 ).

We evaluated the effects of SVZ irradiation (Figures 1A and 1B) on the production of new neurons, by quantifying doublecortin (DCX) staining in the SVZ and OB. DCX is a microtubule-associated protein produced by neuronal progenitors and immature neurons. It can therefore be used as a reliable marker for the quantification of adult neurogenesis [27], [28].

**Figure 2:**
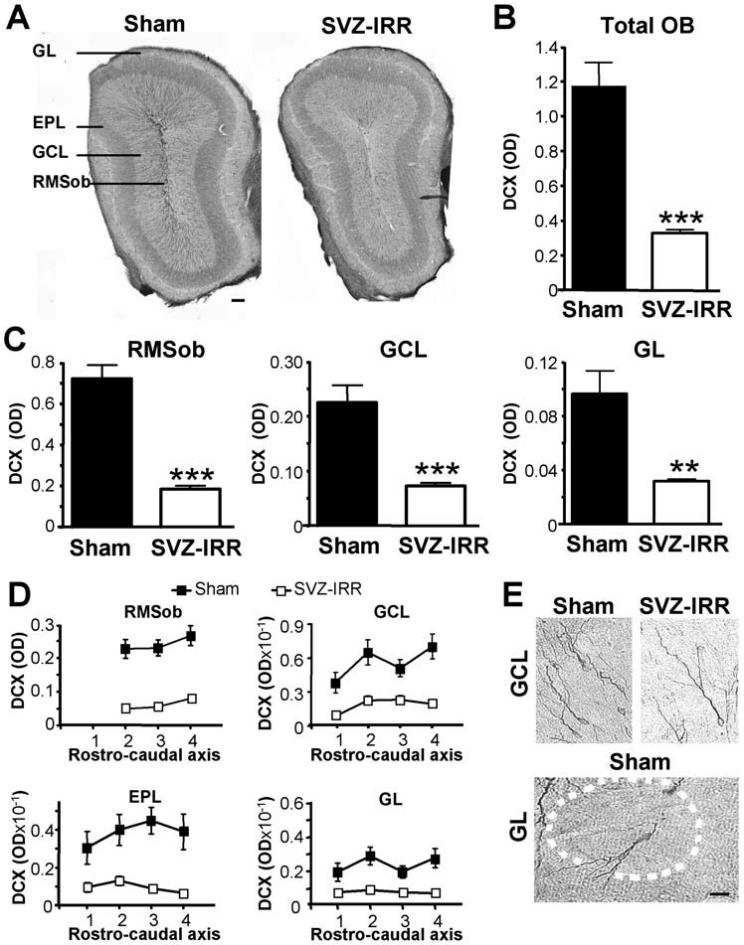
Irradiation decreased the number of DCX^+^ cells in the OB. (A) Representative images showing DCX^+^ cells in coronal sections of the OB, 7 months after SVZ irradiation. The GL, EPL, GCL and RMSob are indicated. (Scale bar: 100 µm). (B and C) Densitometric analysis of DCX immunoreactivity in total OB (B), including the RMSob (C, left), GCL (C, middle) and GL, (C, right) of sham-treated and irradiated mice 7 months after irradiation. OD, optical density. Student's *t* test; *** *p*<0.0001. ** *p*<0.01 (*n* = 12). (D) Densitometric analysis of DCX staining along the rostrocaudal axis of the OB, in sham-treated mice and irradiated mice 7 months after irradiation (*n* = 12). All cell layers along the entire rostrocaudal axis of the OB were equally affected by SVZ irradiation. (E) Immature neurons visualized by DCX staining in the GCL and GL of sham-treated and irradiated mice, 7 months after irradiation. (Scale bar: 20 µm.)

Several months after SVZ irradiation, neurogenesis levels were significantly lower than normal in both the SVZ (Figure 1C and 1D) and OB (Figure 2A and 2B). A complete statistical analysis is provided in the Supplementary Table S1. This finding is consistent with a previous study showing that SVZ progenitor cells are sensitive to irradiation [26]. Levels of DCX immunoreactivity were significantly lower in the SVZ of irradiated mice (about 30% those of sham-treated mice, Figure 1D; DCX optical density for sham treatment: 0.096±0.009, for SVZ-irradiation treatment: 0.03±0.003, *p*<0.0001). Similarly, DCX immunoreactivity throughout the entire OB was 70% weaker in irradiated mice than in sham-treated mice (1.177±0.134 in sham-treated mice; 0.327±0.022 in SVZ-irradiated mice, *p*<0.0001; see Figure 2A and 2B).

All the cell layers in the OB were similarly affected (Figure 2C). Irradiation decreased DCX immunoreactivity in the RMS layer at the core of the OB (RMSob) by 75% (sham-treated: 0.722±0.065 and SVZ-irradiated: 0.185±0.015, *p*<0.0001). Similarly, DCX immunoreactivity in the GCL and GL was 70% lower after irradiation (GCL: 0.224±0.031 in sham-treated, 0.072±0.006 in SVZ-irradiated, *p*<0.0001; GL: 0.096±0.017 in sham-treated, 0.031±0.002 in SVZ-irradiated, *p*<0.01). This pattern was observed along the entire length of the rostrocaudal axis of the OB (Figure 2D). We then used DCX immunoreactivity to assess the number of dendrites and the dendritic morphology of the newly generated cells reaching the OB. These features were not affected by irradiation (Figure 2E, see also Supplementary Figure S1).

**Figure 3:**
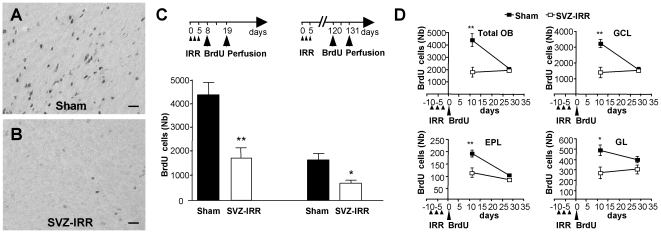
Irradiation reduced the recruitment of new neurons. (A, B) New cells were labeled with BrdU 3 days after the last focal irradiation and survival was determined 11 days later. Photomicrographs show OB coronal sections labeled with BrdU, for sham-treated and irradiated mice. (Scale bar: 30 µm). (C) New cells were labeled with BrdU 8 or 120 days after the first session of irradiation. The mean number of BrdU^+^ cells in the entire OB was determined for sham-treated and irradiated mice 11 days after the final BrdU injection. Student's *t* test; ** *p*<0.01. * *p*<0.05 (*n* = 3–6 mice). (D) BrdU^+^ cell number in the OB, including the GCL, EPL and GL, for sham-treated and irradiated mice, with BrdU injected 8 days after the first session of irradiation. Student's *t* test; ** *p*<0.01. * *p*<0.05 (*n* = 4–6).

We confirmed the lower level of cell proliferation in the irradiated SVZ, by quantifying bromodeoxyuridine (BrdU) staining (Figure 3A and 3B) at two time points after the final irradiation session (Figure 3C). Animals were injected with BrdU three days after the final irradiation session. They were then killed 11 days after BrdU injection (14 days after irradiation). Far fewer BrdU^+^ cells were found in the OB of irradiated mice(60% fewer) than in that of sham-treated mice (4,387±969 BrdU^+^ cells in sham-treated mice, 1,792±859 after irradiation; *p*<0.005). A similar pattern was seen in all layers (for GCL: 3,241±432 and 1,405±697, *p*<0.005; for EPL: 193±14 and 115±19, *p*<0.01; and for GL: 490±95 and 273±120, *p*<0.05, for sham-treated and SVZ-irradiated mice, respectively; Figure 3D).

We then injected mice with BrdU 115 days after the final irradiation session and killed them for analysis 11 days after BrdU injection (Figure 3C). The number of BrdU^+^ cells was decreased to a similar extent in irradiated mice (60% fewer positive cells in irradiated mice; 1,684±276 *vs.* 724±124, *p*<0.05; Figure 3C). As a control, we analyzed the number of BrdU^+^ cells in the hippocampus of SVZ-irradiated mice. The number of positive cells in this part of the brain was similar in irradiated and sham-treated animals (Supplementary Figure S2), demonstrating the confinement of exposure to gamma radiation to the targeted area.

Finally, we analyzed the survival rate of newly generated neurons in the OB, by counting BrdU^+^ cells 31 days after BrdU injection. Consistent with previous studies (*e.g.*, [29]), the number of BrdU^+^ cells halved between day 11 (D11) and D31 in the control OB (*p*<0.005). Similar decreases were observed in the GCL, EPL and GL (Figure 3D). Surprisingly, in sharp contrast to what was observed for the controls, the number of BrdU^+^ cells did not decrease significantly between D11 and D31 (*p*>0.05) in irradiated animals. Thus, local irradiation of the SVZ significantly impaired the recruitment of newly generated OB neurons, and the neurons that actually reached the OB escaped cell apoptosis.

### Spontaneous odorant discrimination was not affected by SVZ irradiation

**Figure 4:**
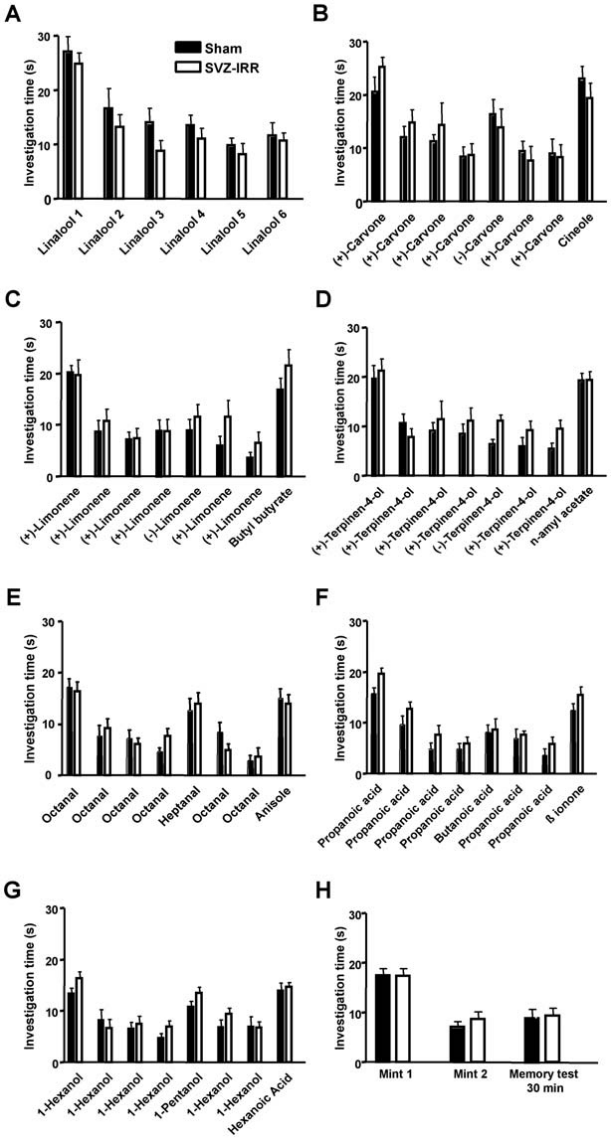
Spontaneous discrimination was not affected by irradiation. Sham-treated and irradiated mice were tested daily, in successive sessions of habituation/dishabituation and memory tests. Histograms indicate the mean time spent investigating an odorant during 90 seconds of exposure, with a two-minute period of rest between consecutive exposures. (A) Habituation with 6 successive exposures to linalool. Both groups showed progressively less interest in investigating the same odorant in repeated exposures, a process called habituation. (B–G) Sessions of habituation (4 successive exposures to the odorants indicated) followed by dishabituation (a single period of exposure to an odorant similar to that used for habituation), recall of habituation (two successive exposures to the odorant used for habituation) and a final dishabituation with a single exposure to a dissimilar odorant. The time spent investigating the test odorant is shown. The extent of dishabituation was similar for the 2 groups: all mice detected even small differences between similar odorants (3B). Neither irradiated nor sham-treated mice could distinguish spontaneously between the limonene and terpinene enantiomers (3C and D). No significant effects of irradiation were observed (Two-way ANOVA; *p*>0.05, *n* = 9–10 mice). (H) 30-minute olfactory memory was not affected by irradiation. A mint odorant was introduced into the cage for five minutes. Two minutes later, the same odorant was introduced again for five minutes. The odorant was introduced into the cage for a final two-minute period after a 30-minute rest period (memory test). Histograms indicate the mean time of investigation. No effect of irradiation was observed (two-way ANOVA; *p*>0.05 *n* = 9–10 mice).

Given the strong effects of irradiation on neuron production, we investigated possible effects on olfaction. As irradiation has transient side effects on the functioning of the mature nervous system due to local inflammation [30], we carried out all behavioral experiments at least two months after irradiation, when inflammation markers had disappeared (data not shown). As a further control, we checked that there was no change in locomotor activity or anxiety levels in irradiated animals (data not shown). Using both non-operant and operant conditioning paradigms, we investigated odorant detection, discrimination and olfactory memory. We first subjected mice to a spontaneous discrimination task involving cross-habituation, to measure their ability to distinguish between different odorants in the absence of associative learning or previous odor reinforcement (Figure 4). Mice were initially trained through six successive exposures to linalool (Figure 4A). Both groups showed a progressive decrease in investigating the same odorant in repeated exposures, a process called habituation. Two-way ANOVA revealed a significant effect of repeated exposure (*p*<0.0001), but no effect of treatment (irradiation) or exposure x treatment interaction (*p*>0.05) was found. Mice were then subjected to a habituation/dishabituation session in which they were exposed to three different odorants every day over a six-day period (Figure 4B–4G). After the fourth sequential exposure to the odorant to which they had been habituated, a similar, but different, odorant was introduced. Mice were exposed to the odorant to which they had been habituated twice more before the introduction of a third odorant from an odorant family different from that to which the first two odorants belonged. We found no difference in the time spent investigating each odorant, for any of the sessions, between sham-treated and irradiated mice (*p*>0.05). Moreover, the extent of dishabituation was similar for the two groups (*p*>0.05): all mice detected even small differences between similar odorants (Figure 4B). Conversely, neither irradiated nor sham-treated mice were able to distinguish spontaneously between the limonene and terpinene enantiomers (Figures 4C and 4D, respectively). A complete statistical analysis is provided in Supplementary Table S2. Thus, spontaneous olfactory discrimination was not affected by SVZ irradiation.

Short-term olfactory memory was studied by exposing mice twice to mint odorant (habituation) and then exposing them to this same odorant again after a 30-minute interval (Figure 4H). Both groups spent less time investigating the odorant during the second and third exposure periods (effect of exposure: *p*<0.0001; irradiation: *p*>0.05, and interaction: *p*>0.05), demonstrating that short-term memory was similar in irradiated mice (tested over a period of 30 minutes) and in controls. Thus, decreasing the number of newly generated neurons reaching the OB has no effect on olfactory discrimination or short-term memory.

### The recognition of social olfactory cues did not require adult OB neurogenesis

All the odorants used for the behavioral tests were artificial. We therefore checked that we had not missed a potential consequence of reducing OB neurogenesis due to our use of synthetic odorants (*i.e.,* ethologically non-relevant molecules). We used a social interaction test to quantify the spontaneous investigation of an unfamiliar mouse by a resident mouse. We found that the two groups of mice spent similar amounts of time investigating the intruder (23.2±2.0 s and 23.5±3.4 s for sham-treated and irradiated mice, respectively; *p*>0.05; see also Supplementary Table S2). Thus, the disruption of adult neurogenesis did not impair the processing of social olfactory information.

### Odorant detection did not depend on adult OB neurogenesis

**Figure 5:**
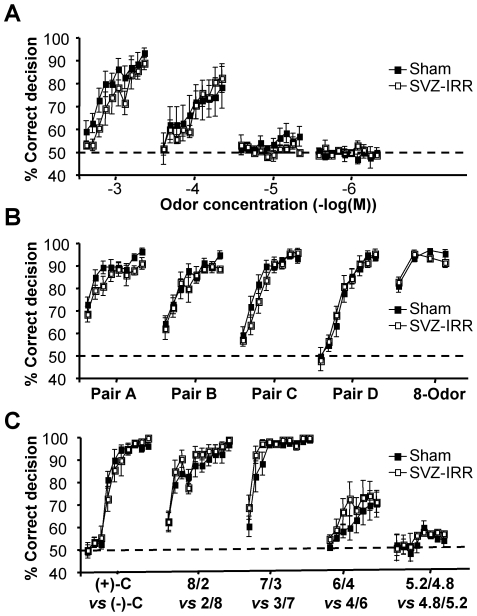
Irradiation has no effect on performance in reinforced discrimination tasks. (A) Odor detection thresholds were not altered by irradiation. Accuracy (% of correct responses) is shown for the detection of successively lower concentrations of (+)-carvone (10 blocks of 20 trials). (+)-carvone was the rewarded (S+) stimulus and the solvent, mineral oil (MO), was the non-rewarded (S-) stimulus. Water-deprived mice were first trained to distinguish between a high concentration of (+)-carvone and MO. They were then subjected to daily blocks of trials in which they were exposed to progressively lower concentrations. Acquisition rate was similar for the 2 groups. A score of 50% corresponds to the success rate expected on the basis of chance alone (dashed line, A–C). No significant differences were observed for irradiated mice (*p*>0.05, *n* = 7). (B) The acquisition of discrimination ability in separate 2-odorant discrimination tasks and performance in the corresponding 8-odorant task were not affected by irradiation. The accuracy of performance in the discrimination tasks is shown as a % of correct responses for 8 blocks of 20 trials for odorant pair A (1% anisole, S+ *vs* 1% cineole, S-), odorant pair B (0.1% n-amyl acetate, S+ *vs* 1% linalool, S-), odorant pair C (1% butanoic acid, S+ *vs* 1% beta-ionone, S-), odorant pair D (1% (+)-limonene, S+ *vs* 1% (+)-carvone, S-) and 4 blocks of 40 trials for 8-odorant tasks. In the two-odorant tests, the stimuli, A, B, C and D (giving eight possible permutations) were introduced in a random order. No effect of SVZ irradiation was observed (two-way ANOVA; *p*>0.05, *n* = 9–10). (C) The ability to distinguish between pairs of mixtures of two odors was not affected by irradiation. Mixtures contained 1% (+)-carvone (indicated by (+)-C or S+) and 1% (−)-carvone (indicated by (−)-C or S-). Five alternating mixtures with different ratios were used and animals were rewarded only when a go-response was observed in the presence of mixtures in which (+)-carvone was the dominant compound. Concentrations (%) of odors are given for the following pairs of mixtures: 8/2 *vs* 2/8: 0.8% (+)-C+0.2% (−)-C (S+) *vs* 0.2% (+)-C+0.8% (−)-C (S-); 7/3 *vs* 3/7: 0.7% (+)-C+0.3% (−)-C (S+) *vs* 0.3% (+)-C+0.7% (−)-C (S-); 6/4 *vs* 4/6: 0.6% (+)-C+0.4% (−)-C (S+) *vs* 0.4% (+)-C+0.6% (−)-C (S-); 5.2/4.8 *vs* 4.8/5.2: 0.52% (+)-C+0.48% (−)-C (S+) *vs* 0.48% (+)-C+0.52% (−)-C (S-). Performance accuracy is shown as a % of correct responses for 10 blocks of 20 trials. No effect of SVZ irradiation was observed (two-way ANOVA; *p*>0.05, *n* = 7).

We hypothesized that the absence of an obvious phenotype in irradiated animals might be due to the use of high concentrations of odorants. We therefore used an automated operant conditioning procedure (a go/no-go test) to investigate olfactory sensitivity, by determining the detection threshold for (+)-carvone, using the descending limits method. (+)-carvone was the rewarded (S+) stimulus, and the solvent, mineral oil, was the unrewarded (S-) stimulus. Water-deprived mice were first trained to distinguish between a high concentration (10^−3^) of (+)- carvone and mineral oil. They were then subjected to daily blocks of trials involving exposure to progressively lower concentrations (10^−4^, 10^−5^ and 10^−6^). Performance accuracy decreased with decreasing concentration for both groups (Figure 5A; *p*<0.05). An analysis of odorant concentration-performance curves for the last block of exposures revealed that both irradiated and sham-treated mice performed the test with an accuracy of more than 80% for concentrations of 10^−3^ and 10^−4^ (+)-carvone (see Supplementary Table S3 for a complete statistical analysis). Furthermore, the rate of successful task completion was similar for the two groups (effect of treatment: *p*>0.05; block of trials: *p*<0.001; interaction: *p*>0.05). By contrast, the accuracy with which both irradiated and sham-treated mice performed the task fell to levels consistent with chance alone (50% correct detection) at 10^−5^ and 10^−6^ (+)-carvone (treatment: *p*>0.05; block of trials: *p*>0.05; interaction, *p*>0.05). The (+)-carvone detection threshold was similar in irradiated and control mice (treatment: *p*>0.05; interaction: *p*>0.05). We therefore conclude that SVZ irradiation does not impair odorant detection.

### Irradiated mice successfully completed two-odorant discrimination tasks

We used the same procedure to explore further the ability of mice to distinguish between different pairs of odorants (Figure 5B). Both groups acquired the ability to distinguish between pairs of odorants in our separate two-odorant discrimination tasks (a complete statistical analysis is provided in Supplementary Table S3). The acquisition rate was similar in the two groups (pair A–D: effect of treatment: *p*>0.05; block of trials: *p*<0.001; interaction: *p*>0.05). Mice from both groups reliably distinguished between all eight odors introduced in a random order within the same session (treatment: *p*>0.05; block of trials: *p*<0.001; interaction: *p*>0.05). Thus, SVZ irradiation does not affect acquisition of the ability to complete two- to eight-odorant discrimination tasks successfully.

### Irradiated mice performed odorant-mixture tasks accurately

Mice were then exposed to more complex problems, using a discrimination task based on binary odorant mixtures. The introduction of mixtures of various proportions of (+)-carvone and (−)-carvone increased the complexity of the task. Five alternating mixtures with different ratios were used and animals were rewarded only when a go-response was observed in the presence of mixtures in which (+)-carvone was the dominant compound (see Supplementary Table S3 for statistical analysis). Performance accuracy varied significantly with the ratio of the mixture (Figure 5C; *p*<0.001). Mice from both groups successfully learned to distinguish between pure odorants (+)-carvone and (−)-carvone and between the following pairs of mixtures: 80%–20% *vs.* 20%–80% and 70%–30% *vs.* 30%–70%. However, performance accuracy was substantially lower (close to the levels expected on the basis of chance alone), in both groups, for distinguishing between 60%–40% and 40%–60% or 52%–48% and 48%–52% mixtures. Thus, the ability to distinguish between mixtures of odorants was similar in the two groups of mice, even for difficult tasks (for pairs of (+)/(−)-carvone mixtures, 8/2 *vs.* 2/8, 7/3 *vs.* 3/7, and 6/4 *vs.* 4/6: effect of treatment, *p*>0.05; block of trials, *p*<0.001; treatment x block interaction, *p*>0.05; for 5.2/4.8-4.8/5.2 mixtures: treatment and block, *p*>0.05; treatment x block interaction, *p*>0.05). SVZ irradiation therefore does not impair the ability to complete difficult olfactory discrimination tasks successfully.

### Long-term olfactory memory was sensitive to SVZ irradiation

**Figure 6:**
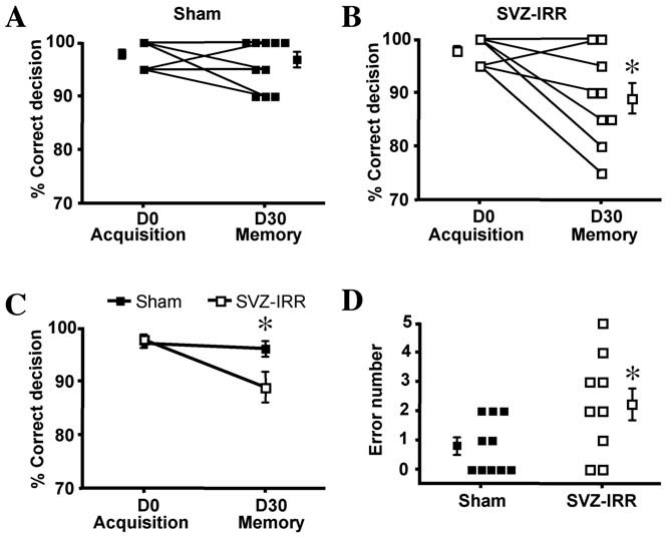
Impaired long-term memory in irradiated mice. Mice underwent 8 blocks of 20 trials every day for 4 days, to train them to distinguish between 1% anisole (rewarded odorant) and 1% cineole (non-rewarded odorant). Mice were tested on the same task after a rest period of one month, in one block of 20 trials, but with no reward given for a correct response. Representative results for experiments performed in triplicate are shown. (A–C) Mean values (%) for correct responses in the last block of training (acquisition) and in the first block of testing (memory test) are shown for each sham-treated (A) and irradiated mouse (B) and for all mice (C). (D) Means of errors in trials 1 to 20 of the memory test session. Two-way ANOVA followed by unpaired or paired Student's *t* tests, as appropriate; * *p*<0.05 (*n* = 9–10).

Finally, we examined the ability of irradiated mice to remember two odorants learned 30 days before. Both groups were first trained to distinguish between two new odorants: anisole (S+) and cineole (S-). The mice were then subjected to the same discrimination task 30 days later. In this second session, no reward was given for correct responses. The lack of reinforcement following the introduction of the S+ stimulus excluded the possibility of an accurate performance by the animals simply reflecting a rapid transfer of training between tasks. We measured the percentage of correct responses for both groups during the last block of the acquisition period and during the first block of the memory task performed 30 days later (Figure 6A–C). For each group, we also calculated the mean number of errors made during the session (Figure 6D). These experiments confirmed that both sham-treated and irradiated mice were able to acquire odor-associated memory in an operant two-odorant task. However, a significant difference in performance was observed between sessions (*p*<0.01), together with a significant interaction between treatment and session (*p*<0.05; a complete statistical analysis of the data is provided in the supplementary Table S4). Performance accuracy was significantly lower in the second session for irradiated mice (*p*<0.05) but not for sham-treated animals (*p*>0.05). Thus, memory of learned odorants was better retained over a one-month period in sham-treated than in irradiated mice. Irradiated animals made significantly more errors in the memory task than sham-treated animals (Figure 6D, *p*<0.05). All errors made during this session were associated with responses triggered by S-, suggesting that the mistakes made by irradiated mice were due to an impaired memory of odors, but not of the go/no-go task procedure. This effect on olfactory memory was replicated with four different odorant pairs (data not shown). Thus, although the ability to detect and discriminate between odorants was not affected by irradiation, irradiated animals remembered odorants less well one month later.

## Discussion

Neurogenesis in the healthy adult brain is principally limited to two systems: the hippocampal dentate gyrus and the SVZ-OB. Its conservation across all mammalian species and tight regulation [5], [31]–[33] suggest that adult neurogenesis may affect behavior. In this study, we investigated the functional consequences for olfaction of reducing adult neurogenesis. We found that impairment of the ongoing recruitment of adult-generated OB neurons altered long-term olfactory memory, but had no effect on odorant detection or the discrimination, learning and recognition of social olfactory cues.

### Focal irradiation of the SVZ reduced the number of newborn neurons in the OB

Adult neurogenesis encompasses cell production, cell fate determination, survival, integration, and the acquisition of functional neuronal properties in the adult brain [5]. Consistent with previous studies [26], [30], DCX staining demonstrated that SVZ irradiation reduced the production of newborn neurons in the SVZ. DCX staining at the time of behavioral testing or seven months after administration of the final dose of radiation revealed a long-lasting effect on OB neurogenesis. Focal irradiation led to a permanent 70% decrease in the number of new neurons produced in the SVZ or integrated into the OB circuit, consistent with most newly generated OB neurons being generated by the SVZ in adults. Further studies are required to identify the types of cell in the SVZ sensitive or resistant to irradiation. It will be particularly interesting to determine whether GFAP-positive “B cells” in the SVZ are resistant to radiation-induced cell death.

Consistent with previous findings, we found that about 50% of newly generated cells in control mice died four weeks after their generation. We also showed, for the first time, that cell survival depended on the overall number of newly produced neurons reaching the bulb: in irradiated animals, only 30% of 11-day-old cells reached the bulb, and all were still alive 19 days later (see Figure 3D). Further immunohistological experiments with other markers of neurons, astroglial and oligodendroglial cells are required for the identification of a possible selection of a subpopulation of radiation- and apoptosis-resistant SVZ/OB cells.

Despite the prolonged survival of the newly generated cells, irradiated animals made more errors than controls in the long-term memory task, suggesting that the continuous recruitment of new neurons, and not the total number of neurons *per se*, is a key element in long-term olfactory memory. Further studies are required to determine whether the surviving cells arising from the irradiated SVZ are functionally different from those produced in the non-irradiated forebrain. Similarly, further studies are required to decipher the mechanisms regulating the survival of newly generated neurons. It is possible that newly generated neurons in the OB compete for survival factors (e.g. trophic factors) just as they compete with existing neurons for many of their synaptic inputs. Such competition would account for the longer survival of new neurons in irradiated animals.

### The production of new neurons for olfaction

The functional relevance of adult neurogenesis remains an unresolved issue in neural stem cell biology. One general strategy used to address this problem involves studying the effects on behavior of inhibiting proliferation in a neurogenic area. In this study, we investigated three types of olfactory function, to assess the effects of reducing adult neurogenesis on odorant detection, discrimination and memory. Both spontaneous and reinforced discrimination tests were used to investigate potential differences in olfactory functions [34]. Irradiated mice exposed to synthetic odorants discriminated between these odorants as efficiently as sham-treated mice. This finding is consistent with previous studies showing that neonatal irradiation or the genetic blockade of adult neurogenesis does not impair olfactory discrimination [11], [35]. However, this result contrasts with previous data obtained in NCAM-knockout mice [21] and BDNF Val66Met knock-in mice [22], both of which displayed disrupted neurogenesis. This discrepancy may be due to the impairment of neurogenesis during embryogenesis rather than the disruption of neurogenesis during adulthood.

Irradiation had no effect on social investigation on the basis of olfactory cues (reviewed in [36]). By contrast, Iwata *et al.* [37] observed social interaction deficits in animals subjected to irradiation of the entire forebrain, leading to various undesirable side effects. For example, irradiated rats displayed abnormal locomotor activity, introducing a potential bias into behavioral tests. No such abnormal behavior was observed in our model, probably due to the more restricted ablation of adult neurogenesis.

Most newly produced neurons remaining in the OB after learning are still present several weeks later and can develop thousands of spines [9], [14], [15], [29]. New neurons may be specifically involved in formation of the synaptic network serving as the structural basis for long-term synaptic changes (*the cell-autonomous hypothesis*). This hypothesis is supported by previous results demonstrating that newly generated neurons are unique in that *1*) they have lower thresholds for synaptic plasticity [38], [39], *2*) they induce “synaptic disquietude” [40], *3*) they increase complexity at the ‘gate to long-term memory’ [41], and *4*) they trigger unique responses during odorant familiarization [42]. Thus, bulbar neurogenesis may increase plasticity in several ways, including the addition of new cells, the structural remodeling of neural circuits, and synaptogenesis, and changes in synaptic strength. All these forms of plasticity are consistent with the neurons generated during adulthood being required for long-term olfactory memory. Alternatively, OB neurogenesis may play an important role in the functioning of pre-existing networks (*the host circuit hypothesis*). The activity of established circuits depends on sensory inputs (*i.e.,* the sensory space) and centrifugal fibers (*i.e.*, the internal state). It is possible that new neurons are the main targets of experience-induced changes in the activity of sensory inputs and/or centrifugal fibers, acting as key elements in the retrieval or recall of memory traces. Further experiments are required to test these two hypotheses specifically.

Our findings suggest that there is a correlation between adult neurogenesis and long-term olfactory memory. Similar correlations also emerged from theoretical studies demonstrating the involvement of adult neurogenesis in memory storage, rather than in perception or learning [43], [44]. Aimone *et al*. also suggested that young neurons facilitate the formation of temporal association in memory [45], and play key role in the encoding of memory [46]. Computer-based studies have indicated a role for constitutive adult neurogenesis in mnesic function, providing a theoretical background for future experimental approaches.

Our data support a causal link between the number of new neurons in the OB and long-term olfactory memory, but we cannot exclude the possibility that another area of the brain is affected by SVZ irradiation and participates in the observed changes in long-term olfactory memory. We also cannot rule out the possibility of changes in the neuronal or synaptic activity of the preexisting OB neurons in irradiated animals. Further studies, involving the selective and reversible inhibition of OB neurogenesis, are required to determine whether there is a genuine causal relationship and investigated the feasibility of attenuating and recovering memory function.

Neurogenesis may allow an increase in the complexity of the network for memory consolidation or long-term adaptation processes during adulthood. Improvements in information processing resulting from the incorporation of newly generated neurons may facilitate olfactory learning and memory formation, consistent with the interdependence between memory performance and the degree of neurogenesis. Previous studies showing a transient role for the OB in memory storage support this notion [47]. The natural replacement of bulbar neurons provides a rationale for the transfer of memory traces out of the bulb. The loss of OB neurons may be programmed to occur after the transfer of traces from these neurons to other parts of the brain. Alternatively, a rejuvenating population of neurons capable of rapidly forming synaptic connections may be highly suitable for the function of the OB in the transient processing of information sent elsewhere for storage. Our findings suggest that new neurons are involved in long-term olfactory memory, consistent with the assumption that new neurons provide unique functions for olfaction [48].

## Materials and Methods

### Animals

We used eight-week-old male C57BL/6J (Janvier, Le Genest-Saint-Isle, France) mice. Animals were housed in groups of four or five and maintained in standard conditions (12 h/12 h light/dark cycle, *ad libitum* access to dry food and water; for olfactometer experiments, animals were subjected to partial water deprivation, as described below) in Pasteur Institute animal care facilities officially registered for experimental studies on rodents (Ministry approval number for animal care facilities: A 75-15-08; approval number 75-585 for animal experimentation). All experimental procedures complied with the European Communities Council Directive of 24 November 1986 (86/609/EEC) and European Union guidelines, and were reviewed and approved by our institutional animal welfare committee.

### Irradiation

Mice were irradiated with a medical Alcyon irradiator (gamma-rays ^60^Co). They were anesthetized with ketamine (75 mg/kg, Merial, Lyons, France) and medetomidine (1 mg/kg, Pfizer, Paris, France) by the intraperitoneal (i.p.) route. They were placed in a stereotaxic frame (Stoelting, Wood Dale, Illinois, USA) and exposed to cranial irradiation or not irradiated (sham-treated). Two lead shields protected the body of the mouse during exposure of the SVZ to gamma rays. The first shield consisted of a 10 cm-thick lead brick with a 12 mm diameter circular hole positioned above the mouse's head. The second lead shield was 5 cm thick, with a rectangular opening of 3×11 mm, corresponding to the area of the SVZ (bregma AP: 1.5 and L: 5.5). The OB, RMS and olfactory epithelium were unaffected by the procedure. Radiation (five Gray) was delivered at a rate of 1 Gray/min on days 1, 3 and 5. After exposure, mice were woken up by i.p. injection of atipamezole (1 mg/kg, Pfizer, Paris, France).

### BrdU injections

Mice were injected i.p. with a DNA synthesis marker, BrdU (75 mg/kg, Sigma-Aldrich, St. Louis, MO). They received four injections, at two-hour intervals, on a single day.

### Immunohistochemistry

Mice were deeply anesthetized with sodium pentobarbital (100 mg/kg, Sanofi, Bagneux, France) and perfused transcardially with a solution containing 0.9% NaCl and heparin (5×10^3^ U/ml, Sanofi-Synthelabo, Le Plessis-Robinson, France) at 37°C, followed by 4% paraformaldehyde (PFA) in cold phosphate buffer (PB), pH 7.3. Brains were dissected out and post-fixed by incubation at 4°C in 4% PFA in PB, overnight for BrdU and for one week for DCX staining. Slices were transferred to phosphate-buffered saline (PBS) and kept at 4°C until use. Immunohistochemistry was carried out on 40 µm-thick free-floating serial coronal sections of the brain cut with a vibrating microtome (VT1000S, Leica, Rueil-Malmaison, France) and collected in 0.2% sodium azide (Sigma) in PBS. Brain sections were washed in PBS and treated with 0.2% Triton X-100, 4% bovine serum albumin (both purchased from Sigma) in PBS for 2 h, to non-specific protein binding and to permeabilize membranes. For BrdU staining, sections were treated with 2 N HCl for 30 minutes at 37°C. BrdU and DCX were detected by incubation with a rat monoclonal anti-BrdU antibody (C18, 1: 200; Immunologicals Direct, UK) or a goat anti-DCX antibody (1∶200; Santa Cruz Biotechnology, Santa Cruz, CA, USA). Labeled cells were detected with a peroxidase-conjugated secondary antibody (ABC system, Vector Laboratories, Inc., Burlingame, CA, USA), using biotinylated donkey anti-rat or horse anti-goat IgG (1∶200, Vector Laboratories) and 3,3′-diaminobenzidine (0.05%) as a chromogen (Sigma).

### Image analysis for BrdU^+^ cell counting

We obtained reconstructed images with a 20× objective for one in every six coronal sections of the OB (six sections in total) for each animal (Compix Imaging; Hamamatsu Photonics, Massy, France). BrdU^+^ cells were automatically counted with a dedicated stereological computer program [49]. The internal and external borders of the GL and GCL were drawn manually and cells detected in the entire layer or in the GL, EPL and GCL were counted. Values are expressed as the mean total BrdU^+^ cell count in six sections of the OB per animal. For hippocampal analysis, values are expressed as the mean total number of BrdU^+^ cells counted manually in eight sections of the dentate gyrus per animal.

### Measurement of optical density

DCX expression was quantified by measuring optical density with a dedicated stereological computer program [49], for one in every six coronal sections of the OB and in equivalent selected sections containing the SVZ. After manual selection of the brain area to be analyzed, the density of staining was calculated by dividing the pixel count by the overall area (pixels per mm^2^).

### Spontaneous discrimination of synthetic odorants

#### Olfactory discrimination

For the olfactory discrimination task, we used a slightly modified version of the habituation–dishabituation test described elsewhere [21]. The test cages were boxes of 36×23.5×13 cm (length×width×height), with two compartments separated by an aluminum partition (holes 0.5 cm diameter). The lower compartment was 2.5 cm high. Animals were exposed to odors by placing a filter paper dish (70 mm diameter, #1440 070; Whatman, Florham Park, NJ, USA), impregnated with 0.4% odorant in 10 µl of odorless mineral oil (odorants and mineral oil obtained from Sigma) in the lower compartment. Four days before the experiment, the animals were familiarized with the test cage and the procedure by exposing them to mineral oil. Mice underwent one session per day. The mouse was placed in the test cage for 10 minutes and exposed to mineral oil for 2 minutes before each session. Mice were trained with six exposures to 0.4% linalool. They were then exposed to odorants as follows:

- Four successive exposures to the first odorant (habituation odor);
- One exposure to a second similar odor;
- Two exposures to the habituation odor;
- One exposure to a dissimilar odorant (dishabituation).

Each exposure lasted 90 seconds. An interval of 2 minutes was left between trials. We recorded the time that the animals spent investigating the odorant for each experiment. Animals were considered to have recognized an olfactory stimulus when they spent significantly less time investigating an odorant introduced into the cage for a second time.

#### Short-term olfactory memory

Mice were exposed to mint odorant twice (5 minutes each), with a two-minute interval between the two exposures. They were then exposed to this odorant again after a rest period of 30 minutes. The time spent investigating the odorant was recorded for each animal.

#### Social interaction

Social interaction was tested in the same test cages used for spontaneous discrimination. Each mouse was tested for 5 minutes with a C57BL/6 mouse of the same age, sex (male) and weight, reared in the same conditions (in the same animal facilities, in similar cages, each containing 4 to 5 mice). Social interaction was measured as the time the test subject (sham-treated or irradiated) spent interacting with the other mouse (interacting social behavior included following the other animal, anogenital sniffing and allogrooming).

#### Olfactory performance in automated olfactometers

Mice were maintained on a 1 ml/day water deprivation diet for 10 days and then trained in a go/no-go discrimination task in Knosys (Bethesda, MD) computer-controlled olfactometers, as previously described [50]. Mice were trained to respond to the presence of an odorant dissolved in mineral oil (positive stimulus, S+) by licking the water delivery tube situated within the odorant sampling port, and to refrain from responding to the presence of another odorant (negative stimulus, S-). These two types of trials were carried out in a modified random order, such that an equal number of each type occurred in each block of 20 trials and one type of trial did not occur more than three times consecutively. A response in an S+ trial and an absence of response in an S– trial were scored as correct. Accuracy was scored for each block of 20 trials. Mice underwent a session of eight to 10 blocks of trials per day. All odorants were diluted in mineral oil and their concentrations are given as the dilution of the odorant in the saturator bottles.

#### Odorant detection threshold

Mice were trained, in the air dilution olfactometer, to detect successively lower concentrations of (+)-carvone diluted in mineral oil. Each concentration was given for 10 blocks of 20 trials each day. In each session, (+)-carvone vapor served as the S+ stimulus and mineral oil served as the S– stimulus. The concentrations of (+)-carvone used in these tests were 0.001, 0.0001, 0.00001 and 0.000001%.

#### Olfactory discrimination tasks

Mice were trained in a series of two-odorant discrimination tasks using the eight-channel olfactometer. Each mouse was subjected to eight blocks of 20 trials for each of the following tasks:

- Task 1: S+ was 1% anisole and S- was 1% cineole.
- Task 2: S+ was 0.1% n-amyl acetate and S- was 1% linalool.
- Task 3: S+ was 1% butanoic Acid and S- was 1% beta-ionone.
- Task 4: S+ was 1% (+)-limonene and S– was 1% (+)-carvone. Mice not fulfilling the performance criterion of 90% correct responses in two successive 20-trial blocks underwent further training in daily 200-trial sessions until this level of performance was reached.
- Task 5: Eight-odorant discrimination. Upon completing tasks 1–4, each mouse was given additional training, in which they were exposed to the eight odorants used in tasks 1–4 in two blocks of 40 trials. Stimuli were introduced in a modified random order such that, within each block of 40 trials, mice were exposed to the S+ and S– stimuli five times each.

#### Odorant mixture discrimination tasks

Mice were trained to distinguish 1% (+)-carvone from (−)-carvone in 10 blocks of 20 trials (Task 1). Then they were given 10 blocks of 20 trials for each of the following two-odorant mixture tasks:

Task 2: S+ was 0.8% (+)-carvone +0.2% (−)-carvone and S- was 0.2% (+)-carvone +0.8% (−)-carvone.

Task 3: S+ was 0.7% (+)-carvone +0.3% (−)-carvone and S- was 0.3% (+)-carvone +0.7% (−)-carvone.

Task 4: S+ was 0.6% (+)-carvone +0.4% (−)-carvone and S- was 0.4% (+)-carvone +0.6%(−)-carvone.

Task 5: S^+^ was 0.52% (+)-carvone +0.48% (−)-carvone and S- was 0.48% (+)-carvone +0.52% (−)-carvone.

#### Long-term memory test

We followed the memory test procedure described by Bodyak and Slotnick [50], with minor modifications. Mice were given four daily training sessions of eight blocks of 20 trials for a two-odorant task (S+ was 1% anisole and S- was 1% cineole). Mice were then left for 32 days in their home cages, with partial water deprivation for the last 10 days. They were given no water on day 31. The following day, each mouse was subjected to a 20-trial memory test for the two-odorant task. No reinforcement was given for correct responses in this session. Mice therefore received no feedback concerning whether their responses were correct or incorrect.

#### Statistical analysis

All data are expressed as means±SEM. Statistical analyses were carried out with Prism software (Graphpad Software, San Diego, USA). For immunohistochemistry data, we used Student's *t*-test. Behavioral data were analyzed with parametric methods: unpaired Student's *t-*tests were used to compare the two groups, with unpaired observations to assess social interaction. Spontaneous discrimination and olfactometer data were analyzed by standard two-way analysis of variance (ANOVA) followed by unpaired or paired Student's *t*-tests, as appropriate.

## Supporting Information

Figure S1 Irradiation did not alter the morphology of DCX+ cells reaching the OB. Dendrites were counted for DCX+ cells in the GCL, EPL and GL of sham and irradiated mice, 7 months after irradiation. P>0.05 with Student's t-test (n = 25 from 5 random cells analyzed per OB layer and from 5 mice per group).
(2.75 MB EPS)

Figure S2 Focal SVZ irradiation inhibited the recruitment of new neurons in the OB but not in the hippocampus. New cells were labeled with BrdU 3 days after the final focal irradiation and survival was evaluated 11 days later. The mean number of BrdU+ cells in the GCL of the OB and in the dentate gyrus of the hippocampus was determined for sham and irradiated mice. ** indicates p<0.01, Student's t test, n = 6 mice per group.
(4.54 MB EPS)

Table S1 Complete statistical analysis on neurogenesis data.
(0.04 MB RTF)

Table S2 Complete statistical analysis on spontaneous discrimination.
(0.08 MB RTF)

Table S3 Complete statistical analysis for operant conditioning.
(0.11 MB RTF)

Table S4 Statistical analysis on 2-odor memory test.
(0.03 MB RTF)
