# Supplementary material for: Cellular and Behavioral Effects of Cranial Irradiation of the Subventricular Zone in Adult Mice
Source: PLoS One. 2009 Sep 15;4(9):e7017. doi: 10.1371/journal.pone.0007017 (PMC2737283; doi:10.1371/journal.pone.0007017)
Supplement: Table S1 — Complete statistical analysis on neurogenesis data. (0.04 MB RTF) [file pone.0007017.s003.rtf]

Supplemental Table S1 : Complete statistical analysis on neurogenesis data

Experiment	Assessment and animal number	Statistical Test	Comparison	Statistics	p	Fig.	
DCX expression	SVZ,n=6 per group	Student's t test	Unpaired t-test	T=7.114	<0.0001	1D	
	OB, n=12 per group	Student's t test	Unpaired t-test	T=6.274	<0.0001	2B	
	RMSob, n=12 per group	Student's t test	Unpaired t-test	T=8.055	<0.0001	2C	
	GCL, n=12 per group	Student's t test	Unpaired t-test	T=4.68	<0.0001	2C	
	GL, n=12 per group	Student's t test	Unpaired t-test	T=3.696	<0.005	2C	
BrdU + cells	OB, 16 D after IRR, n=6 per group	Student's t test	Unpaired t-test	T=3.893	<0.005	3C-D	
	OB, 115 D after IRR, n=3 per group	Student's t test	Unpaired t-test	T=3.17	<0.05	3C	
	GCL, 16 D after IRR, n=6 per group	Student's t test	Unpaired t-test	T=4.288	<0.005	3D	
	EPL, 16 D after IRR, n=6 per group	Student's t test	Unpaired t-test	T=3.271	<0.01	3D	
	GL, 16 D after IRR, n=6 per group	Student's t test	Unpaired t-test	T=2.815	<0.05	3D	
	OB of sham mice, n=5-6 	Student's t test	Unpaired t-test	T=3.801	<0.005	3D	
	OB of IRR mice, n=4-5 	Student's t test	Unpaired t-test	T=0.279	>0.5	3D	
	OB, n=4-5 per group	Student's t test	Unpaired t-test	T=0.637	>0.5	3D	
