# Supplementary material for: Cellular and Behavioral Effects of Cranial Irradiation of the Subventricular Zone in Adult Mice
Source: PLoS One. 2009 Sep 15;4(9):e7017. doi: 10.1371/journal.pone.0007017 (PMC2737283; doi:10.1371/journal.pone.0007017)
Supplement: Table S2 — Complete statistical analysis on spontaneous discrimination. (0.08 MB RTF) [file pone.0007017.s004.rtf]

Supplemental Table S2 : Complete statistical analysis on spontaneous discrimination 

Experiment	Fig.	Assessment 	Statistical test	Comparison	Statistics	Df	p	
Spontaneous olfactory discrimination	4A	Investigation time (exp 1)	2-way ANOVA	Presentation 	F=13.51	5	<0.0001	
				Treatment	F=3.91	1	>0.05	
				Interaction	F=0.208	5	>0.05	
	4B	Investigation time (exp 2)	2-way ANOVA	Presentation 	F=9.524	7	<0.0001	
				Treatment	F=0.053	1	>0.05	
				Interaction	F=0.660	7	>0.05	
	4C	Investigation time (exp 3)	2-way ANOVA	Presentation 	F=11.70	7	<0.0001	
				Treatment	F=3.582	1	>0.05	
				Interaction	F=0.493	7	>0.05	
	4D	Investigation time (exp 4)	2-way ANOVA	Presentation 	F=13.39	7	<0.0001	
				Treatment	F=3.717	1	>0.05	
				Interaction	F=0.727	7	>0.05	
	4E	Investigation time (exp 5)	2-way ANOVA	Presentation 	F=16.67	7	<0.0001	
				Treatment	F=0.010	1	>0.05	
				Interaction	F=0.768	7	>0.05	
	4F	Investigation time (exp 6)	2-way ANOVA	Presentation 	F=19.16	7	<0.0001	
				Treatment	F=7.728	1	>0.05	
				Interaction	F=0.352	7	>0.05	
	4G	Investigation time (exp 7)	2-way ANOVA	Presentation 	F=12.82	7	<0.0001	
				Treatment	F=3.096	1	>0.05	
				Interaction	F=0.621	7	>0.05	
Spontaneous odor memory at 30 min	4H	Investigation time	2-way ANOVA	Presentation 	F=25.34	2	<0.0001	
				Treatment	F=0.269	1	>0.05	
				Interaction	F=0.198	2	>0.05	
Social interaction	ND	Time spent in social behaviour	Student's t test	Unpaired t-test	T=0.097	17	>0.05	
Animal number : n=10 sham, n=9 irradiated ; Df : Degrees of freedom ; exp : experiment ; ND : Not shown
