# Supplementary material for: Cellular and Behavioral Effects of Cranial Irradiation of the Subventricular Zone in Adult Mice
Source: PLoS One. 2009 Sep 15;4(9):e7017. doi: 10.1371/journal.pone.0007017 (PMC2737283; doi:10.1371/journal.pone.0007017)
Supplement: Table S3 — Complete statistical analysis for operant conditioning. (0.11 MB RTF) [file pone.0007017.s005.rtf]

Supplemental Table S3 : Complete statistical study for operant conditioning
Experiment	Fig.	Assessment 	Statistical test	Comparison	Statistics	Df	p	
Odor sensivity	5A	Correct decision (10-3 dilution)	2-way ANOVA	Block 	F=9.504	9	<0.0001	
				Treatment	F=3.355	1	>0.05	
				Interaction	F=0.510	9	>0.05	
		Correct decision (10-4 dilution)	2-way ANOVA	Block 	F=3.759	9	<0.0001	
				Treatment	F=0.033	1	>0.05	
				Interaction	F=0.201	9	>0.05	
		Correct decision (10-5 dilution)	2-way ANOVA	Block	F=0.617	9	>0.05	
				Treatment	F=3.878	1	>0.05	
				Interaction	F=0.377	9	>0.05	
		Correct decision (10-6 dilution)	2-way ANOVA	Block 	F=0.319	9	>0.05	
				Treatment	F=1.486	1	>0.05	
				Interaction	F=0.617	9	>0.05	
		Correct decision (Last block)	2-way ANOVA	Concentration	F=37.54	3	<0.0001	
				Treatment	F=0.332	1	>0.05	
				Interaction	F=0.943	3	>0.05	
Odor discrimination	5B	Correct decision (pair A)	2-way ANOVA	Block 	F=13.17	7	<0.0001	
				Treatment	F=2.512	1	>0.05	
				Interaction	F=0.226	7	>0.05	
		Correct decision (pair B)	2-way ANOVA	Block 	F=15.97	7	<0.0001	
				Treatment	F=1.3	1	>0.05	
				Interaction	F=0.541	7	>0.05	
		Correct decision (pair C)	2-way ANOVA	Block	F=52.92	7	>0.05	
				Treatment	F=0.813	1	>0.05	
				Interaction	F=0.201	7	>0.05	
		Correct decision (pair D)	2-way ANOVA	Block 	F=54.75	7	>0.05	
				Treatment	F=0.336	1	>0.05	
				Interaction	F=0.309	7	>0.05	
		Correct decision (8-odor)	2-way ANOVA	Block	F=23.88	7	<0.0001	
				Treatment	F=1.715	1	>0.05	
				Interaction	F=2.614	7	>0.05	
Odor –mixture tasks	5C	Correct decision (Carvones)	2-way ANOVA	Block 	F=80.94	9	<0.001	
				Treatment	F=0.015	1	>0.05	
				Interaction	F=0.284	9	>0.05	
		Correct decision (8/2 vs 2/8)	2-way ANOVA	Block 	F=13.55	9	<0.001	
				Treatment	F=2.868	1	>0.05	
				Interaction	F=0.446	9	>0.05	
		Correct decision (7/3 vs 3/7)	2-way ANOVA	Block	F=4.279	7	<0.001	
				Treatment	F=1.733	1	>0.05	
				Interaction	F=0.613	7	>0.05	
		Correct decision (6/4 vs 4/6)	2-way ANOVA	Block 	F=5.753	7	<0.001	
				Treatment	F=2.423	1	>0.05	
				Interaction	F=0.013	7	>0.05	
		Correct decision (5.2/4.8 vs 4.8/5.2)	2-way ANOVA	Block	F=2.029	7	>0.05	
				Treatment	F=3.554	1	>0.05	
				Interaction	F=0.518	7	>0.05	
		Correct decision (last block)	2-way ANOVA	Mixture	F=98	4	<0.001	
				Treatment	F=0.058	1	>0.05	
				Interaction	F=0.297	4	>0.05	
Animal number : n=7-10 per group ; Df : Degrees of freedom
