# Supplementary material for: Cellular and Behavioral Effects of Cranial Irradiation of the Subventricular Zone in Adult Mice
Source: PLoS One. 2009 Sep 15;4(9):e7017. doi: 10.1371/journal.pone.0007017 (PMC2737283; doi:10.1371/journal.pone.0007017)
Supplement: Table S4 — Statistical analysis on 2-odor memory test. (0.03 MB RTF) [file pone.0007017.s006.rtf]

Supplemental Table S4 : Statistical analysis on 2-odor memory test 
                


Assessment
	Fig.	Statistical Test	Comparison	Statistics	Df	p	
Correct decision 	6C	Two-way ANOVA	Session 	F=8.8	1	<0.005	
			Treatment	F=3.61	1	>0.05	
			Interaction	F=5.6	1	<0.05	
	6C	Unpaired Student's t test	Memory task : Sham vs irradiated	T=2.286	17	<0.05	
	6A	Paired Student's t test	Sham : memory vs acquisition task	T=0.61	9	>0.05	
	6B	Paired Student's t test	Irradiated : memory vs acquisition task	T=2.98	8	<0.05	
Error number 	6D	Student's t test	Irradiated vs sham	T=2.28	17	<0.05	
n=10 sham mice, n=9 irradiated mice ; Df : Degrees of freedom
